# Editorial: Multi-omics data integration: key to thoroughly understanding the immune system

**DOI:** 10.3389/fcell.2023.1207318

**Published:** 2023-05-09

**Authors:** Cristina Jiménez, María Hernández-Sánchez, Jacques J. M. van Dongen, Paula Díez

**Affiliations:** ^1^ Hematology Department, University Hospital of Salamanca (HUS/IBSAL), CIBERONC and Cancer Research Institute of Salamanca-IBMCC (USAL-CSIC), Salamanca, Spain; ^2^ Department of Biochemistry and Molecular Biology, Universidad Complutense de Madrid, Madrid, Spain; ^3^ Translational and Clinical Research Program, Cancer Research Center (IBMCC, University of Salamanca—CSIC), Cytometry Service, NUCLEUS, Department of Medicine, University of Salamanca and Institute of Biomedical Research of Salamanca (IBSAL), Salamanca, Spain; ^4^ Department of Immunology, Leiden University Medical Center (LUMC), Leiden, Netherlands; ^5^ Health Research Institute of Asturias (ISPA) and Asturias Central University Hospital (HUCA), Oviedo, Spain

**Keywords:** multi-omics, immune system, cancer, proteomics, transcriptomics, immune infiltration, biomarker

The integration of platforms such as genomics, transcriptomics and proteomics has been acknowledged as a promising strategy for the in-depth characterization of complex cell systems and/or diseases. These omics platforms are now also helping us understand human immunity better. The immune system is a highly sophisticated network that maintains tissue homeostasis throughout the body. Thus, any disturbance in the immune system directly impacts diseases or indirectly drives malignant cell development. In this Research Topic, six manuscripts aim to describe the immune system by integrating different omics approaches, investigating the immunity functioning and its involvement in disease or the efficacy of therapeutical drug responses.

A first study, from Staal et al., outlined the main steps and critical points to successfully perform single-cell RNA sequencing (scRNASeq) analysis on rare immune cells within the T-cell development pathways in the human thymus. The authors also considered the integration with existing datasets and proteomics data obtained by flow cytometry (FC). It is well known that scRNASeq is an optimal approach to investigating complex heterogeneous samples and cell types. However, as the authors pointed out, the correlation between RNA and protein levels is usually poor, hampering the full cell characterization when only one platform is used. Therefore, the investigation of the transcriptomes and proteomes at the single-cell level would be highly beneficial for the comprehensive understanding of cell features. Thus, their study design proposed the inclusion of i) the enrichment of rare populations, ii) the cell isolation, iii) the selection of the most appropriate sequencing protocol by taking into account the number of cells and sequencing depth required, iv) the data analysis including quality control and normalization steps, integration and regression methods, and dimensionality reduction, v) the integration with existing datasets and vi) the functional validation using FC as a proteomics approach.

The second study, from Yin et al., performed RNASeq and whole-genome sequencing on peripheral blood samples from recovered 25-45 years-old COVID-19 patients vs*.* healthy controls (age- and gender-matched). In this research, the authors identified significant changes in the abundances of 639 transcripts and 18,516 DNA-methylated regions. These alterations were associated with the prolonged overreaction of the immune system to SARS-CoV-2 infection and processes such as stress response and metabolic functions. Moreover, the significant number of transposable elements aberrantly activated in COVID-19 patients was positively correlated with increased disease severity. Therefore, the study of the transcriptome and DNA methylome of COVID-19 patients seems to be helpful for the determination of the long-term effects of COVID-19 disease.

In the third study, by Duan et al., a thorough investigation of the ligamentum flavum hypertrophy (LFH) condition was performed. The authors analyzed microarray-derived gene expression data using bioinformatics tools for enrichment (GEO, KEGG), protein-protein interaction (PPI) and immune cell infiltration (CIBERSORT algorithm) and Friends analyses. Validation of several transcripts and proteins (EGFR, IL-6, IL-10, LEP, TNF-α) was performed by quantitative real-time PCR and immunohistochemistry, respectively. As a result, 1,530 differentially expressed genes were identified as being mainly associated with the PI3K-Akt and cytokine activity-related signaling pathways. Moreover, the authors reported activated CD4 memory T cells and naïve B cells as new immune infiltrating cells for the LFH condition.

The other three manuscripts included in this Research Topic focused on the study of the immune system´s involvement in cancer. Yuan et al *.* Conducted a pan-cancer study aiming to elucidate the oncogenic role of the mini-chromosome maintenance 2 (MCM2) protein. They defined the expression level of this protein, its genetic mutation rate and immune infiltration, among others, combining genomics, transcriptomics and proteomics analyses. Their results showed a significant upregulation of MCM2 in 33 types of human cancers, especially in melanoma, which correlated with a poor prognosis and a high degree of immune infiltration in the tumor sites. Altogether, this report highlighted MCM2 as a potential biomarker for cancer diagnosis and prognosis as well as a good candidate for cancer immunotherapy.

Focusing on papillary thyroid carcinoma (PTC), Shih et al. analyzed 27 PTC transcriptomics datasets for the identification of new prognostic biomarkers. Their bioinformatics study revealed an enrichment in the tumoral samples in proteins related to cell-matrix remodeling and transcriptional dysregulation. Also, differentially expressed microRNAs linked to immune response modulation and cell proliferation were uncovered. Additionally, the authors simulated the interaction between those enriched proteins and some bioactive compounds from an indigenous medicinal plant (*Antrodia camphorata*) revealing that the antcin compound (steroid-like phytochemical) displayed the highest binding efficacy towards, e.g., ETV5 and fibronectin. In sum, this study showed the potential applicability of targeting the described PTC-enriched proteins with therapeutic purposes.

Finally, Li et al. investigated the link between six glycosylation regulators, tumorigenesis and immunotherapy in gastric malignancies using the expression data from –2,000 patients. Glycosylation scores (Glyc. score) of the marker genes were calculated for each patient revealing that patients with high glycosylation levels had a poor prognosis and increased immune cell infiltration. The authors suggested using this glycosylation scoring system to predict the treatment response in gastric cancer patients.

In conclusion, this Research Topic provided multidisciplinary omics investigations related to the function and role of the immune system from different perspectives (immune cell infiltration, development, modulation and response to therapies) using and integrating data from diverse omics approaches.

